# Laparotomic vs. laparoscopic myomectomy: surgical outcomes from a tertiary center retrospective study

**DOI:** 10.3389/fsurg.2025.1728370

**Published:** 2026-02-09

**Authors:** Figen Efe Çamili, Tuba Bozhüyük Şahin, Ezgi Tolu Cenk, Selim Afşar, Gürhan Güney, Mine İslimye Taşkın

**Affiliations:** 1 Department of Obstetrics and Gynecology, Faculty of Medicine, Balikesir University, Balikesir, Türkiye; 2 Department of Obstetrics and Gynecology, Balıkesir Atatürk City Hospital, Balikesir, Türkiye

**Keywords:** laparoscopy, laparotomy, myomectomy, postoperative outcomes, uterine myoma

## Abstract

**Objective:**

This study aimed to retrospectively analyze myomectomy cases performed in our clinic using laparotomic and laparoscopic techniques, and to compare the effects of both surgical approaches on various clinical and surgical outcomes.

**Materials and methods:**

Patient records of myomectomy operations performed between 2015 and 2025 at the Department of Obstetrics and Gynecology, Balıkesir University Faculty of Medicine, were reviewed. A total of 213 patients were included, comprising 140 laparoscopic and 73 laparotomic cases. The data such as patient age, number and size of removed myomas, preoperative and postoperative hemoglobin levels, postoperative additional analgesic requirements, length of hospital stay and complication rates will be analyzed to evaluate the advantages of each method in terms of patient comfort and surgical efficacy.

**Results:**

The mean age of patients undergoing laparoscopic myomectomy was significantly higher than those in the laparotomic group (*p* < 0.001). The laparoscopic group demonstrated a significantly shorter hospital stay compared to the laparotomic group (*p* < 0.001). Preoperative and postoperative hemoglobin levels did not differ significantly between the groups. The number of removed myomas was similar; however, the mean myoma diameter was significantly larger in the laparotomic group (*p* < 0.001). Postoperative opioid use was significantly higher in the laparotomic group (*p* = 0.01). Larger and more numerous myomas were independently associated with a higher likelihood of laparotomy over laparoscopy (*p* < 0.001). Among laparoscopic cases, only four required conversion to laparotomy (%2,9) and a single bladder injury was observed.

**Conclusion:**

Laparoscopic myomectomy provides considerable advantages over laparotomic myomectomy, including reduced hospital stay and lower postoperative analgesic requirements. While laparotomy remains preferable for larger myomas, laparoscopic approaches yield comparable outcomes in terms of hemoglobin levels and complication rates. With appropriate patient selection, laparoscopic myomectomy is a preferred surgical method due to its positive impact on patient recovery, comfort and overall surgical efficacy.

## 1 Introduction

Uterine fibroids are the most common cause of pelvic masses in women and are often detected incidentally without symptoms. Although 40% of cases are asymptomatic, these findings are generally associated with discomforting symptoms such as heavy menstrual bleeding, intermenstrual bleeding, abdominal distension, pelvic pain, constipation and urinary incontinence as a result of pressure on surrounding organs (1, 2). In addition, it can cause recurrent pregnancy loss and infertility due to implantation failure (3, 4).

The treatment generally preferred worldwide is usually surgical, involving either the removal of fibroids while preserving the uterus or a hysterectomy. The treatment method is determined based on factors such as the number and size of the fibroids, the patient's age, desire for pregnancy and clinical condition. In addition, factors such as the surgeon's experience, hospital facilities and patient preference also play an important role in this choice. Surgical options include conventional laparotomy, minilaparotomy, hysteroscopy, laparoscopy, Vnotes and robot-assisted methods (5–8).

Laparoscopic procedures are being favored more often because they are associated with reduced postoperative pain, shorter hospitalization, quicker recovery and better cosmetic outcomes. However, in the presence of large, multiple or deep-seated myomas, the laparotomy approach is still an important treatment option. While both methods have different advantages and disadvantages, comparing surgical outcomes is important for selecting the most appropriate surgical method for the patient.

In our study, we aim to compare cases of myomectomy performed using laparotomy and laparoscopy methods in our clinic in terms of parameters such as age, number and type of myomas, postoperative need for additional analgesics and length of hospital stay. In this way, we aim to evaluate the effects of different surgical approaches on patient outcomes and contribute to clinical practice in terms of appropriate patient selection.

## 2 Materials and methods

Myomectomy cases performed at the Department of Obstetrics and Gynecology, Balıkesir University Faculty of Medicine between January 2015 and January 2025 were identified by reviewing hospital records. Patients with incomplete or insufficient data in hospital records, those under 18 years of age, patients with a confirmed or suspected diagnosis of uterine malignancy, those who underwent myomectomy with a different surgical intervention and those who had myomectomy performed during cesarean section were excluded from the study. A total of 213 uterine myoma cases meeting the inclusion criteria, including 140 laparoscopic (LS) and 73 laparotomic(LT) procedures, were retrospectively analyzed.

All surgical procedures were performed by two experienced gynecologists and one gynecologic oncologist. The study examined and recorded patient age, number and size of myomas, preoperative and postoperative (8th hour) hemoglobin levels, postoperative additional analgesic requirements (type, dose, duration), length of hospital stay (days), and postoperative complication status.

Statistical analyses were conducted using Statistical Package for Social Sciences (SPSS) version 29 (IBM Corp., Armonk, N.Y.; USA). The Shapiro–Wilk test was used to assess the normality of continuous variables. For variables that followed a normal distribution, comparisons between the laparoscopic and laparotomic groups were performed using the Student's *t*-test. For non-normally distributed data, the Mann–Whitney *U*-test was used. For multivariate analysis, binomial logistic regression analysis was used. A *p*-value of less than 0.05 was considered statistically significant.

### 2.1 Ethics approval and informed consent

This study was approved by the Balıkesir University Faculty of Medicine Health Research Ethics Committee. (Date:2/9/2025, Decision No:2025/6-18). The study was conducted in accordance with the principles of the 1975 Helsinki Declaration, as revised in 2000. Informed consent was obtained from all subjects involved in the study.

## 3 Results

Demographic and surgical data are summarized in Table 1. The mean age of patients who underwent LS and LT myomectomy was 41.6 ± 6.3 and 38.2 ± 5.1, respectively, and was significantly higher in the laparoscopic group (*p* < 0.001). Myoma diameter was significantly larger in the LT group (8.23 ± 3.59 cm) compared to the LS group (6.03 ± 2.20 cm) (*p* < 0.001). Although the number of myomas removed was slightly higher in the LT group (1.44 ± 0.53 vs. 1.23 ± 0.42), the difference was not statistically significant.

**Table 1:** Comparison of demographic and surgical characteristics between laparoscopic and laparotomic myomectomy cases.

Variables	Laparoscopic myomectomy (*n* = 140)	Laparotomic myomectomy (*n* = 73)	*P* value*
Age_a_ (years)	41.6 ± 6.3	38.2 ± 5.1	**<0** **.** **001**
Myom diameter^a^ (cm)	6.03 ± 2.20	8.23 ± 3.59	**<0** **.** **001**
Myom counts^a^ (number)	1.23 ± 0.42	1.44 ± 0.53	0.98
Preop hb^a^ (g/dL)	12.24 ± 1.44	12.15 ± 1.20	0.67
Postop hb^a^ (g/dL)	11.04 ± 1.24	11.14 ± 1.17	0.63
Hospital stay^a^ (days)	2.06 ± 0.75	2.71 ± 1.18	**<0** **.** **001**
Paracetamol^a^ (doses)	0.42 ± 0.78	0.49 ± 0.69	0.50
NSAI^a^ (doses)	0.58 ± 1.30	0.34 ± 0.80	0.15
Opiate^a^ (doses)	0.16 ± 0.50	0.37 ± 0.63	**0** **.** **01**

NSAIDs, non-steroidal anti-inflammatory drugs.

a Mean ± SD.

* Student's *t*-test. Bold values indicate a statistically significant difference (*p* < 0.05).

Preoperative and postoperative hemoglobin levels were similar between groups. Preoperative hemoglobin levels were 12.24 ± 1.44 g/dL and 12.15 ± 1.20 g/dL with no significant difference (*p* = 0.95). Postoperative hemoglobin levels also showed no significant difference (*p* = 0.11).

There was a statistically significant difference in hospital stay between the LS (mean: 2.06 ± 0.75 days) and LT (mean: 2.71 ± 1.18 days) groups (*p* < 0.001), with shorter stays in the LS group.

In terms of postoperative analgesic requirements, opioid use was significantly higher in the LT group (mean: 0.37 ± 0.63) than in the LS group (mean: 0.16 ± 0.50) (*p* < 0.001). Paracetamol and non-steroidal anti-inflammatory drugs (NSAID) use did not differ significantly between groups.

Conversion to laparotomy occurred in only 4 of 140 laparoscopic cases (2.9%). A single case of bladder injury was observed in the LS group; no complications were reported in the LT group.

**Figure 1:**
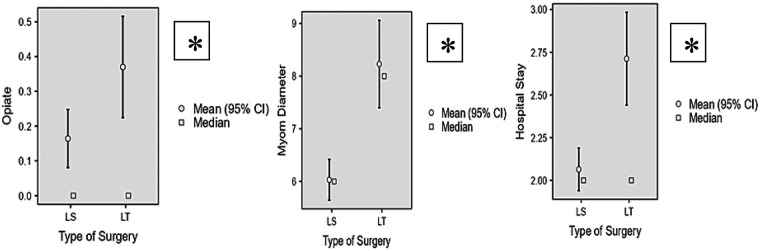
Plots demonstrating postoperative opioid use, myoma diameter and hospital stay across surgical groups (LS vs. LT). LS, laparoscopy; LT, laparotomy. *statistically significant (*p* < 0,05).

Postoperative opioid use, myoma diameter and hospital stay differed between surgical approaches as illustrated with plots in Figure 1 (*p* < 0,05).

Binomial logistic regression analysis revealed that myoma size and number were the strongest independent predictors of surgical method; larger and more numerous myomas were more likely to be treated with laparotomy rather than laparoscopy (*p* < 0.001). Hospital stay also showed a significant association with surgical type (*p* = 0.007) (Table 2).

**Table 2:** Predictors of surgical approach in logistic regression (laparoscopy vs. laparotomy).

Predictor	β (Estimate)	SE	*p*-value
Hospital stay	−0.56	0.21	**0**.**007**
Myoma diameter	−0.33	0.09	**<0**.**001**
Preoperative Hb	0.18	0.26	0.48
Postoperative Hb	−0.16	0.29	0.59
Paracetamol use	0.02	0.24	0.92
NSAID use	0.32	0.21	0.13
Opiate use	−0.34	0.36	0.35
Myom counts	1.48	0.45	**<0**.**001**

Estimates represent the log odds of “Type of Surgery=Laparoscopy “ vs. “Type of Surgery=Laparotomy “ Bold values indicate a statistically significant difference (*p* < 0.05).

## 4 Discussion

In this retrospective study, we found that patients undergoing laparoscopy had shorter hospital stays and lower postoperative opioid requirements. Patients in the LT group had significantly larger and more numerous myomas, suggesting that open surgery is preferred for more complex cases. Despite differences in surgical approach, pre- and postoperative hemoglobin levels were similar, indicating comparable intraoperative blood loss. Although there was no difference in the use of paracetamol and NSAIDs, the lower opioid requirement in the LS group supports that the minimally invasive approach enhances postoperative comfort. Opioid requirement can be explained by differences in surgical trauma level, incision size or tissue manipulation. The approximately one-day difference in hospital stay may seem small; however, it is clinically meaningful for early mobilization and a faster return to daily activities.

Our findings are consistent with previous randomized studies and meta-analyses (9–14). In contrast to the meta-analysis by Giannini et al., our study found that the mean age of patients in the LS group was higher than that in the LT group (9). This indicates that, when selecting the surgical approach, the size and number of fibroids may play a more decisive role than patient age.

Minimally invasive surgery is safe and effective in appropriate cases, providing the advantage of reduced blood loss, particularly in patients with small and limited numbers of fibroids (11). Unlike previous reports, our study did not observe a significant difference in hemoglobin drop between the groups, which may be explained by surgical experience, patient characteristic and study size. Previous meta-analyses have reported that the operative time for LS is generally longer than for LT (9–13). However, in our study, these data were not available and thus no comparison could be made.

Capozzi et al., in their meta-analysis, also demonstrated that LS offers an advantage in terms of postoperative complications (13). Similarly, we observed a low complication rate. In our study, the occurrence of a complication in only one case precluded statistical comparison.

In a recent meta-analysis by İbrahim et al., long-term outcomes were also evaluated. Pregnancy rates were found to be higher in the LS group, while no differences were observed in delivery mode or miscarriage rates (14). Although our perioperative findings are similar to those reported in this meta-analysis, no conclusions can be drawn regarding long-term fertility outcomes, as our study did not assess them.

In our study, the conversion rate to open surgery was 2.9%, which was low compared to the range reported in the literature. Recent studies have reported laparoscopic conversion rates ranging from 0.8% to 17% depending on factors such as the size and number of fibroids and the surgeon's experience (15–17). The relatively low conversion rate observed in our study may reflect the extensive laparoscopic training of our surgical team and careful patient selection. These findings are consistent with reports suggesting that experienced surgeons and careful preoperative planning can minimize the need to convert from laparoscopic myomectomy to open myomectomy.

The main factors limiting the applicability of laparoscopic myomectomy are adequate visualization during the procedure, effective management of intraoperative bleeding and potential complications that may arise during the removal of the myoma from the body. Fibroids can range in size from a few millimeters to several centimeters, and as their volume increases, laparoscopic visualization may be limited. Therefore, careful preoperative assessment of the size, number and location of fibroids is crucial. Although some older series have suggested size or number-based limits for laparoscopic myomectomy, there is no universally accepted cutoff value in the current literature. The literature also reports that experienced surgeons can safely remove myomas up to 20 cm in size laparoscopically (18–20).

A critical aspect of the analysis is that the size and number of fibroids can independently affect surgical outcomes as well as the surgical approach. This potential imbalance may have contributed to slightly worse outcomes in the open surgery group.

Considering all these studies and meta-analyses, both the literature and our study have shown that minimally invasive surgery provides perioperative benefits without compromising reproductive outcomes when patient selection is appropriate. The choice of surgical approach should be personalized based on fibroid characteristics, the surgeon's expertise and fertility considerations. Although robotic myomectomy, one of the minimally invasive surgical techniques, has gained attention in recent years due to the advantages it offers in terms of surgeon ergonomics and surgical comfort, all patients in our study underwent laparoscopic and laparotomic myomectomy; this approach provided significant benefits in terms of both cost and time.

### 4.1 Study limitations

One of the strengths of our study is the relatively large number of cases collected over a ten-year period, which enabled us to compare perioperative outcomes in detail. However, due to the retrospective design, there is a possibility of selection and information bias; the data were obtained from hospital records and are not based on prospective follow-up. In addition, incomplete documentation in some variables and the inability to fully control potential confounding factors such as patient characteristics or coexisting comorbidities may limit the interpretation of the results. The study was conducted at a single university hospital and the generalizability of the findings to other institutions or different patient populations may be limited. All gynecologists participating in this study had received comprehensive training in laparoscopic surgery, and their surgical experience was comparable. Moreover, long-term postoperative outcomes, such as fertility, myoma recurrence or quality of life were not evaluated. These issues require further investigation through prospective randomized studies.

## 5 Conclusion

Laparoscopic myomectomy provides shorter hospital stays, reduced postoperative opioid use and faster recovery compared to open surgery, with similar safety. The choice of surgical approach should be tailored to the patient, taking into account the characteristics of the myoma and the surgeon's experience.

## Data Availability

Relevant data supporting the findings can be provided by the corresponding author upon reasonable request. Requests to access these datasets should be directed to Figen Efe Çamili, efefigenefe@gmail.com.
